# Aggravation of Reflux Finding Score (RFS) after thyroidectomy

**DOI:** 10.1371/journal.pone.0254235

**Published:** 2021-07-26

**Authors:** Hyung-Joon Yoon, Hee Ryung Kim, Chang Myeon Song, Ji Young Lee, You Hern Ahn, Kyung Tae

**Affiliations:** 1 Department of Otolaryngology-Head and Neck Surgery, College of Medicine, Hanyang University, Seoul, Republic of Korea; 2 Department of Radiology, College of Medicine, Hanyang University, Seoul, Republic of Korea; 3 Department of Internal Medicine, College of Medicine, Hanyang University, Seoul, Republic of Korea; Brigham and Women’s Hospital, Harvard Medical School, UNITED STATES

## Abstract

Laryngopharyngeal reflux (LPR) has been suggested as a possible cause of post-thyroidectomy syndrome. However, the pathophysiology and relationship between thyroidectomy and LPR have not been well investigated. We aimed to evaluate the correlation between thyroidectomy and LPR by assessing changes in LPR-related symptoms and laryngoscopic findings before and after thyroidectomy. Ninety-five patients who underwent thyroidectomy with or without central neck dissection were included. The reflux finding score (RFS) and reflux symptom index (RSI) were investigated one day before surgery and two, four, six, and twelve months after surgery. The RFS scores increased significantly after thyroidectomy and decreased to the preoperative level 12 months after surgery. The RSI scores increased after surgery and decreased gradually by 12 months postoperatively, although it was not statistically significant. The RSI and RFS scores improved with the administration of proton pump inhibitors. In conclusion, LPR-related laryngoscopic findings were exacerbated after uncomplicated thyroidectomy. Further studies using pH-monitoring and esophageal manometry are required to investigate the possible deterioration of LPR itself and the UES pressure after thyroidectomy.

## Introduction

Hoarseness, foreign body sensation in the throat, cough, and swallowing problems are commonly presented after thyroidectomy, even in the absence of recurrent laryngeal nerve (RLN) palsy [1–5]. These symptoms, including voice impairment, throat symptoms, and swallowing disorder after uncomplicated thyroidectomy, are associated with post-thyroidectomy syndrome. Although the etiology and pathogenesis of post-thyroidectomy syndrome are not clearly defined, possible causes may include laryngotracheal fixation, the external branch of superior laryngeal nerve (EBSLN) injury, cricothyroid muscle damage, endotracheal intubation, injury to the fine anastomotic branches connecting the RLN and EBSLN, and psychotic reaction [2, 4–6].

It has been suggested that laryngopharyngeal reflux (LPR) also plays a role in patients with post-thyroidectomy syndrome [7, 8]. LPR is a retrograde flow of gastric and duodenal contents into the pharynx or larynx above the upper esophageal sphincter (UES). LPR presents in about 50% of patients with voice or throat disorders [9–12]. Laryngopharyngeal reflux disease (LPRD) is different from gastroesophageal reflux disease (GERD) in its pathophysiology and symptoms. Heartburn and regurgitation are common in GERD but rare in LPRD [12, 13]. LPR irritates and injures the larynx and pharynx, causing morphological changes and various symptoms, such as chronic cough, sore throat, globus, hoarseness, and swallowing difficulties, similar to symptoms of post-thyroidectomy syndrome [14–16].

Although LPR has been suggested as one of the causes of various throat symptoms after thyroidectomy, the pathogenesis and relationship between LPR and thyroidectomy have not been thoroughly investigated. This study aimed to evaluate the correlation between thyroidectomy and LPR by assessing changes in LPR-related symptoms and findings before and after thyroidectomy.

## Materials and methods

### Patients

We analyzed 95 patients who underwent thyroidectomy with or without central neck dissection between January 2013 and February 2014 and who completed the serial assessment of LPR-related symptoms and fiberoptic laryngoscopy. The exclusion criteria were < 18 years old, vocal mucosal lesions such as vocal nodules, polyps, Reink’s edema, and sulcus vocalis, pre- and postoperative RLN or EBSLN palsy, and systemic neural movement disorder. Patients who underwent lateral neck dissection simultaneously with thyroidectomy or revision thyroidectomy and patients with upper aerodigestive tract invasion, postoperative hematoma, or distant metastasis were excluded. Patients with a history of neck irradiation or previous neck surgery were excluded. Videostroboscopy (Xion, Berlin, Germany) was performed one day before and the day after surgery to examine the presence of vocal mucosal lesions and the injury to the RLN and the EBSLN.

Initially, we enrolled 167 patients during the study period. Of the 167 patients, 72 patients were excluded from the study due to postoperative transient RLN palsy (n = 5), concomitant vocal cord lesions (n = 9), performing completion thyroidectomy (n = 4), postoperative hematoma (n = 1), invasion of the trachea (n = 1), previous history of neck surgery (n = 3), and refusal to complete the assessment of postoperative LPR-related symptoms and serial laryngoscopy (n = 49). Finally, 95 patients were included in the analysis. The written informed consent was obtained from all patients. The study protocol was approved by the Institutional Review Board of Hanyang University Hospital.

### Assessment of Reflux Symptom Index (RSI) and Reflux Finding Score (RFS)

The reflux symptom index (RSI) and reflux finding score (RFS) suggested by Belafsky et al. were used as LPR parameters in this study [13, 14]. The RSI and RFS were evaluated prospectively on the day before surgery and two, four, six, and 12 months after thyroidectomy in the outpatient clinic. LPRD was defined as an RSI score ≥ 13, or an RFS score ≥ 7 [13, 14].

The RSI is a self-administered questionnaire that contains nine items to evaluate patients’ subjective LPR-related symptoms and severity. The nine items of the RSI were hoarseness, vocal fatigue, excessive throat clearing, globus pharyngeus, coughing after eating or lying down, chronic cough, throat mucous or postnasal drip, dysphagia, and heartburn or chest pain. Each item of the RSI was scored from zero to five according to the severity of symptoms [13].

Fiberoptic flexible laryngoscopy was used to assess RFS. The RFS was scored by the presence of eight items of the laryngoscopic findings, including subglottic edema, ventricular obliteration, erythema or hyperemia, vocal fold edema, diffuse laryngeal edema, posterior commissure hypertrophy, granuloma or granulation tissue, and endolaryngeal mucus, and the scores ranged up to 26 [14]. Laryngoscopic findings for the RFS scores were judged in all patients by a single author (HJY) blindly when conducting the study to minimize inter-examiners’ bias.

### Statistical analysis

All data were coded and entered into the Statistical Package for the Social Sciences (SPSS) software (version 18.0, SPSS Inc., Chicago, IL, USA). A paired t-test and Wilcoxon signed-rank test were used to compare the paired data pre- and postoperatively. Categorical binary variables were compared using the chi-square test. A P-value of less than 0.05 was considered statistically significant.

## Results

The clinicopathologic characteristics of the patients and the surgical extent are presented in Table 1. Of the 95 patients, 73 were female, and 22 were male. The mean age was 48 ± 12 years. Regarding pathology, 89% were malignant, and 11% were benign. The mean tumor size was 10.6 ± 10.2 mm. Total thyroidectomy was performed in 60% of the patients, and thyroid lobectomy was performed in 40%. Central neck dissection was performed in 92% of the patients.

**Table 1:** The clinicopathologic characteristics of patients and surgical extent.

Characteristic	Number of patients (%) (n = 95)
Sex	
Female	73 (77%)
Male	22 (23%)
Age (years)	47.8 ± 12.1
Pathology	
Benign	10 (11%)
Malignant	85 (89%)
Tumor size (mm)	10.6 ± 10.2
Extent of thyroidectomy	
Total thyroidectomy	57 (60%)
Lobectomy	38 (40%)
Central neck dissection	87 (92%)

The changes in RFS and RSI before and after surgery are presented in Table 2. The RFS scores increased significantly after surgery by six months and decreased to a level that was not different from the preoperative score at 12 months postoperatively. The RSI scores tended to increase after surgery and gradually decrease until 12 months after surgery, although it was not statistically significant before and after surgery.

**Table 2:** The Reflux Finding Score (RFS) and Reflux Symptom Index (RSI) before and after thyroidectomy (n = 95).

**Reflux finding score**	Mean ± SD	*P*-value^a^
Pre-op	6.95 ± 9.57	
Post-op 2 months	10.71 ± 6.99	<0.001
Post-op 4 months	10.45 ± 5.33	<0.001
Post-op 6 months	10.77 ± 6.29	0.005
Post-op 12 months	9.28 ± 5.56	0.094
**Reflux symptom index**	Mean ± SD	*P*-value^*a*^
Pre-op	5.84 ± 5.83	
Post-op 2 months	7.09 ± 7.76	0.421
Post-op 4 months	6.31 ± 7.03	0.328
Post-op 6 months	6.01 ± 6.94	0.840
Post-op 12 months	5.53 ± 7.63	0.276

Abbreviation: SD, standard deviation; Pre-op, preoperative; Post-op, postoperative.

^a^compared with the preoperative score

Based on the RSI and RFS scores, LPRD was diagnosed in 25 (26.3%) patients preoperatively. The number of patients with LPRD increased to 37 (38.9%) and 29 (30.5%) at two and four months postoperatively, respectively. The RFS and RSI scores were compared before and after surgery in the two subgroups according to the presence and absence of preoperative LPRD. In 25 patients with LPRD preoperatively, the RFS score increased significantly at two months after surgery and decreased to the preoperative level four months postoperatively. The RSI scores did not change significantly before and after surgery (Table 3). In the subgroup of 70 patients without LPRD, 17 patients met the criteria of LPRD by RFS or RSI at least one more time after surgery. The RFS scores increased at two, four, and six months after surgery and decreased to the preoperative level 12 months postoperatively. The RSI scores did not differ before and after thyroidectomy (Table 3).

**Table 3:** The Reflux Finding Score (RFS) and Reflux Symptom Index (RSI) before and after thyroidectomy according to the presence of Laryngopharyngeal Reflux Disease (LPRD) preoperatively.

**Patients with LPRD (n = 25)**					
**RFS**	Mean ± SD	*P*-value^a^	**RSI**	Mean ± SD	*P*-value^a^
Pre-op	11.04 ± 4.05		Pre-op	12.28 ± 6.85	
Post-op 2 months	15.54 ± 5.32	0.007	Post-op 2 months	12.04 ± 9.46	0.723
Post-op 4 months	13.06 ± 5.89	0.289	Post-op 4 months	10.70 ± 8.87	0.512
Post-op 6 months	11.73 ± 5.22	1.000	Post-op 6 months	11.22 ± 8.93	0.522
Post-op 12 months	13.33 ± 6.89	0.416	Post-op 12 months	11.79 ± 11.36	0.559
**Patients without LPRD (n = 70)**					
**RFS**	Mean ± SD	*P*-value^a^	**RSI**	Mean ± SD	*P*-value^a^
Pre-op	4.35 ± 1.52		Pre-op	3.49 ± 2.95	
Post-op 2 months	8.03 ± 3.25	<0.001	Post-op 2 months	5.29 ± 6.21	0.095
Post-op 4 months	8.00 ± 3.34	0.002	Post-op 4 months	4.69 ± 5.46	0.230
Post-op 6 months	6.87 ± 2.22	0.005	Post-op 6 months	3.95 ± 4.63	0.448
Post-op 12 months	6.77 ± 3.88	0.098	Post-op 12 months	3.61 ± 4.73	0.471

Abbreviation: SD, standard deviation; RFS, reflux finding score; RSI, reflux symptom index; LPRD, laryngopharyngeal reflux disease; Pre-op, preoperative; Post-op, postoperative

^a^compared with the preoperative score

Of the 95 patients, 64 (67.4%) were treated with proton pump inhibitors (PPIs) according to the presence of symptoms and their preferences for PPI. Of the 64 patients who were administered a PPI, 34 had LPRD, and 30 did not have LPRD. The PPIs used in this study were lansoprazole, rabeprazole, and esomeprazole. In about 70% of the patients, PPI medication was started within 2.5 months after the operation using a standard dose, and the mean duration of PPI medication was 5.86 ± 4.02 months (range, 1–12 months).

In 64 patients administered PPIs, the RFS and RSI scores decreased at two and four months after PPI administration, especially in patients with LPRD (Table 4).

**Table 4:** Changes in the Reflux Finding Score (RFS) and Reflux Symptom Index (RSI) before and after the administration of a Proton Pump Inhibitor (PPI) (n = 64) according to the presence of Laryngopharyngeal Reflux Disease (LPRD).

**Patients with LPRD (n = 34)**					
**RFS**	Mean ± SD	*P*-value^a^	**RSI**	Mean ± SD	*P*-value^a^
Before PPI	13.71 ± 5.10		Before PPI	13.35 ± 9.58	
After 2 months PPI	8.77 ± 4.15	<0.001	After 2 months PPI	8.27 ± 6.93	0.002
After 4 months PPI	7.11 ± 3.32	<0.001	After 4 months PPI	7.11 ± 6.77	<0.001
**Patients without LPRD (n = 30)**					
**RFS**	Mean ± SD	*P*-value^a^	**RSI**	Mean ± SD	*P*-value^a^
Before PPI	5.38 ± 1.59		Before PPI	4.05 ± 3.84	
After 2 months PPI	5.00 ± 4.06	0.064	After 2 months PPI	3.59 ± 3.27	0.102
After 4 months PPI	4.00 ± 1.23	0.263	After 4 months PPI	2.46 ± 2.83	0.005

Abbreviation: SD, standard deviation; RFS, reflux finding score; RSI, reflux symptom index; PPI, proton pump inhibitor; LPRD, laryngopharyngeal reflux disease

^a^compared with the scores before medication

## Discussion

LPR is suggested to be closely associated with various voice and swallowing disorders before and after thyroidectomy. Some studies have reported that LPR is related to throat symptoms in patients with a thyroid nodule, even before thyroidectomy [7, 17]. Fiorentino et al. found that 88% of patients with a thyroid nodule or goiter presenting compressive symptoms had LPR on videofluoroscopic swallowing study, preoperatively [7]. In addition, all patients with LPR preoperatively showed LPR postoperatively as well on videofluoroscopic swallowing study [7]. Moreover, a few papers reported that throat symptoms after thyroidectomy were improved with the administration of PPI [18].

In this study, we analyzed the RFS and RSI before and after thyroidectomy for up to one year in 95 patients. The RFS scores increased significantly after thyroidectomy and returned to the preoperative level 12 months postoperatively. In addition, the RSI scores tended to increase after surgery, before gradually decreasing until 12 months after surgery, although this finding was not statistically significant. The deterioration of LPR-related laryngoscopic findings after thyroidectomy occurred in both groups regardless of the presence of preoperative LPRD. In addition, the RSI and RFS improved significantly with the administration of PPI. The results of this study might suggest that thyroidectomy aggravates LPR postoperatively, and the deterioration of LPR plays a role in post-thyroidectomy symptoms. However, aggravation of reflux-related signs and symptoms could be associated with other unknown causes.

Thyroidectomy might worsen the anti-reflux defense mechanism and aggravate LPR, although a clear etiology and pathogenesis of LPR aggravation after thyroidectomy has not been determined yet. Peristaltic motility of the esophagus and the tone of UES are important defense mechanisms to protect the upper aerodigestive tract against gastric acid reflux [16]. Scerrino et al. evaluated the pressures of UES and lower esophageal sphincter (LES), and esophageal motility and coordination using esophageal manometry performed preoperatively and at 30–45 days after thyroidectomy [19]. They reported that the UES pressure was reduced by approximately 25% after thyroidectomy, although it remained within the normal range. However, the LES pressure and esophageal body mobility did not change before and after surgery.

In particular, the UES tone and transient UES relaxations are involved in the pathophysiology of LPR, although the trigger mechanisms have not been clearly identified [16, 20]. If the UES function is impaired, gastric acid refluxes into the laryngeal area and causes various symptoms of LPRD.

The UES is influenced by the EBSLN and RLN [21, 22]. The EBSLN supplies the cricothyroid muscle, and approximately 20% of the EBSLN innervates the pharyngeal inferior constrictor muscle, which is composed of the UES [22]. The RLN has motor fibers innervated to all the intrinsic laryngeal muscles and sensory branches of the larynx. The posterior branches of the RLN are anastomosed with fibers of the EBSLN and innervate the cricopharyngeal muscle [21, 22]. It might be hypothesized that thyroidectomy could lead to unrecognized injury to the EBSLN or the fine anastomotic branches of the laryngeal nerves, innervating the UES, pharynx, and larynx [1, 8, 9]. Damage to the fine anastomotic branches of the laryngeal nerves could lead to decreased UES tone and aggravation of LPR, even in the absence of EBSLN and RLN injuries. However, this is a hypothesis, and further studies are necessary.

This study has several limitations. First, it was not a randomized controlled study. Therefore, selection bias is inevitable. Second, we did not examine whether LPR itself is aggravated after surgery using 24 h pH-monitoring. Additionally, we did not measure the UES pressure. Therefore, we cannot confirm whether the patient’s throat symptoms and LPR-related laryngoscopic findings were derived from LPR itself or other causes besides LPR, although the RSI and RFS are used as standard parameters of LPR in clinics. It is difficult to perform 24 h pH-metry and esophageal manometry in reality because they are invasive procedures, and a number of patients refuse to undergo them. Third, no randomization was performed between the groups with or without PPI administration. Therefore, there is a bias in the interpretation of the effect of PPI medication. Fourth, electromyography was not performed routinely to confirm injury to the EBSLN due to its invasiveness, although EBSLN palsy was evaluated using videostroboscopy. Fifth, endotracheal intubation for general anesthesia might irritate the larynx and pharynx and cause throat symptoms. However, the effect of endotracheal intubation usually resolves within one to two weeks [4]. Therefore, the effect of endotracheal intubation is limited in this study.

Despite these limitations, it is noteworthy that this study revealed significant aggravation of the RFS score after thyroidectomy. It might suggest that LPR plays a role in post-thyroidectomy syndrome, although we did not confirm LPR deterioration in this study.

## Conclusions

LPR-related laryngoscopic findings were exacerbated after uncomplicated thyroidectomy. Further studies using pH-monitoring and esophageal manometry are required to investigate the possible deterioration of LPR itself and the UES pressure after thyroidectomy.
